# Ultra-rare renal diseases diagnosed with whole-exome sequencing: Utility in diagnosis and management

**DOI:** 10.1186/s12920-021-01026-6

**Published:** 2021-07-03

**Authors:** Jiwon Jung, Joo Hoon Lee, Young Seo Park, Go Hun Seo, Changwon Keum, Hee Gyung Kang, Hajeong Lee, Sang Koo Lee, Sang Taek Lee, Heeyeon Cho, Beom Hee Lee

**Affiliations:** 1 Department of Pediatrics, Asan Medical Center Children’s Hospital, University of Ulsan College of Medicine, 88, Olympic-ro 43-gil, Songpa-gu, Seoul, 05505 Republic of Korea; 2 3Billion, Inc., Seoul, South Korea; 3 Department of Pediatrics, Seoul National University Children’s Hospital, Seoul, Republic of Korea; 4 Division of Nephrology, Department of Internal Medicine, Seoul National University Hospital, Seoul, Republic of Korea; 5 Division of Nephrology, Department of Internal Medicine, Asan Medical Center, University of Ulsan College of Medicine, Seoul, Republic of Korea; 6 Department of Pediatrics, Samsung Changwon Hospital, Sungkyunkwan University School of Medicine, Changwon, Republic of Korea; 7 Department of Pediatrics, Samsung Medical Center, Sungkyunkwan University School of Medicine, Seoul, Republic of Korea; 8 Medical Genetics Center, Asan Medical Center Children’s Hospital, University of Ulsan College of Medicine, Seoul, Republic of Korea

**Keywords:** Ultra-rare disease, Genetic renal disease, Whole-exome sequencing, Genetic diagnosis

## Abstract

**Background:**

This study aimed to use whole-exome sequencing (WES) to diagnose ultra-rare renal diseases and the clinical impact of such an approach on patient care.

**Methods:**

Clinical, radiological, pathological, and genetic findings were reviewed in the patients and their family members.

**Results:**

Nine patients from nine unrelated Korean families were included in the study and evaluated. WES identified eight different conditions in these patients, i.e., autosomal dominant tubulointerstitial kidney disease associated with *UMOD* mutation; recurrent urinary stones associated with *APRT* deficiency; Ayme-Gripp syndrome associated with *MAF* mutation; short rib-thoracic dysplasia associated with *IFT140* mutation; renal coloboma syndrome associated with *PAX2* mutations; idiopathic infantile hypercalcemia associated with *CYP24A1* mutation; and hypomagnesemia associated with *TRPM* mutation. Eleven different mutations, including seven novel mutations, were identified, i.e., four truncating mutations, six missense mutations, and one splice-acceptor variant. After genetic confirmation, strategies for the management of the following: medications, donor selection for renal transplantation, and surveillance for extra-renal manifestations were altered. In addition, genetic counseling was provided for the patients and their family members with respect to family member screening for affected but yet unidentified patients and future reproductive planning.

**Conclusion:**

As WES can effectively identify ultra-rare genetic renal diseases, facilitate the diagnosis process, and improve patient care, it is a good approach to enable a better understanding of ultra-rare conditions and for the establishment of appropriate counseling, surveillance, and management strategies.

## Background

More rare diseases are being rapidly identified and are beginning to be considered as independent disease entities. The definition of “rare disease” may differ among nations or organizations, but generally, a disease is generally considered “rare” if it affects less than 1 person in 1500–2000, and an ultra-rare disease has a prevalence of less than 1 person in 50,000 [1].

Accordingly, information regarding rare diseases with renal involvement is increasing. Although most of these diseases manifest during childhood, some patients may be left undiagnosed until adulthood because of the extreme rarity and late-onset of these conditions, or physician unfamiliarity. Diagnosis of these conditions during the early clinical stage is important because proper surveillance and management can halt disease progression and improve the quality of life in affected patients [2].

Traditional diagnostic strategies have some limitations with respect to the early diagnosis of rare renal diseases with a genetic basis. Our strategy, described as the “phenotype to genotype” approach, requires extraction of the core renal phenotype (i.e., glomerular, tubular, tubulointerstitial, vascular, embryological, and cystic), family member evaluation, and identification of extra-renal manifestations such as vision and hearing [3]. After histopathological evaluation of the involved tissues and relevant biochemical tests, candidate genes are selected for disease confirmation. This approach is time- and labor-intensive, and a successful diagnosis is dependent on clinical suspicion by the physician.

Conversely, with the advent of massive parallel sequencing techniques, such as whole-exome sequencing (WES), which have reduced the time, cost, and efficiency of genetic diagnosis, an opposite diagnostic strategy, or the “genotype to phenotype” paradigm, has emerged and facilitated the diagnostic process [3]. Clinical and genetic evaluations in parallel, can enable physicians to re-evaluate the clinical relevance of the genetic defect with respect to the patient’s phenotype and to confirm the diagnosis indicated by the genetic test.

In the current study, we used WES to diagnose patients with ultra-rare renal diseases, and then evaluated the clinical utility of the genetic diagnosis and its impact on patient management and outcomes and to enhance our understanding of ultra-rare renal diseases with a genetic basis.

## Methods

### Patients

From April 2018 to January 2020, 46 patients were referred to the Medical Genetics Center, Asan Medical Center, Seoul, Korea, for the evaluation of possible underlying genetic renal diseases. Among these, 9 patients diagnosed with an ultra-rare renal disease were included.

Medical records were reviewed for family history, clinical, laboratory, laboratory, and genetic findings. The study was approved by the Institutional Review Board of the Asan Medical Center, Seoul, Korea with a waiver of informed consent for a retrospective, de-identified data collection, and analysis (2018-0574, 2018-0180 and 2020-0839).

### Analysis of genetic alterations

Genomic DNA was isolated from peripheral blood or buccal swab samples. WES was performed using genomic DNA. All exons of all human genes (approximately 22,000) were captured using a SureSelect kit (Version C2; Agilent Technologies, Inc., Santa Clara, CA, USA). The captured genomic regions were sequenced using a NovaSeq platform (Illumina, San Diego, CA, USA). Data analyses of raw genome sequences included alignment to the reference sequence (NCBI genome assembly GRCh37; accessed in February 2009). The mean depth of coverage was 100-fold, with 99.2% coverage higher than tenfold. Variant calling, annotation, and prioritization were performed as previously described, and a software program called EVIDENCE developed by 3 billion Inc., Seoul, Korea was used to prioritize variants based on ACMG guideline and the phenotype of each patient [4]. EVIDENCE, an automated computational framework provided variant filtration, classification, prioritization of variants based on multiple computational programs [5], and calculated similarity score independently developed to assess the similarity between the phenotype of each patient and the phenotype predicted by prioritized variants. Sanger sequencing was performed for variants identified by exome sequencing in patients, and a number of in silico analyses were used for the evaluation of functional effect of missense mutations. (Polyphen – 2; http://genetics.bwh.harvard.edu/pph2/index.shtml, SIFT; Sorting Intolerant From Tolerant, https://sift.bii.a-star.edu.sg, PROVEAN; http://provean.jcvi.org/index.php, and InterVar; http://wintervar.wglab.org/).

## Results

During the study period, 9 patients from 9 unrelated families were diagnosed with 7 unique genetic renal diseases associated with 11 gene mutations. The clinical features of the patients are summarized in Table 1.

**Table 1:** Clinical features and genetic alterations of the patients

Pt No	Sex	Phenotype and symptoms	Age at WES (yr)	Renal function	Affected gene	Nucleotide change	Amino acid change	Inheritance	ACMG classification	Reference	Incidence (number of pts)
1	F	Renal insufficiency with hyperuricemic crisis, family history of ESRD of unknown etiology	59	ESRD	*UMOD*	c.626G > T	p.Gly209Val	AD	LP	New ^†^	unknown
2	M	Recurrent ureter stone since the age of 30 with renal insufficiency	41	ESRD	*APRT*	c.G294A	p.Trp98*	AR	P	[14]	Unknown, 1/5000–1/10000
3	F	Dysmorphic face with cleft palate, short stature, scoliosis, microscopic hematuria with renal insufficiency from glomerular mesangiolysis	20	CKD Stage2	*MAF*	c.185C > T	p.Thr62Met	AD	P	New^†^	up to 21
4	M	ESRD at 4 years old with unknown etiology, short stature with a narrow chest, dense skull, family history of same clinical phenotype	18	ESRD	*IFT140*	c.2650C > T c.4309G > A	p.Arg884Trp p.Glu1437Lys	AR	VUS VUS	Found in the general population	up to 20
5	M	Early-onset renal insufficiency with small kidneys	11	CKD Stage 3	*PAX2*	c.124_139del	p.Val42Argfs*36	AD	P	New^†^	up to 60
6	M	Early-onset renal insufficiency with small kidneys	10	CKD Stage 2	*PAX2*	c.686-1G > T	Splice site variant	AD	P	New^†^	up to 60
7	F	Nephrocalcinosis, hypercalciuria with persistent nephrocalcinosis	9	Normal	*CYP24A1*	c.376C > T	p.Pro126Ser	AR	VUS	New^†^	unknown
8	F	Neonatal hypocalcemia, hypomagnesemia with tetany	7	Normal	*TRPM6*	c.1421A > G c.4917_4918delAA	p.Tyr474Cys p.Lys1639Ansfs*4	AR	LP P	New^†^	unknown
9	F	Both renal hypoplasia with multiple cysts, anuria after 17 days of birth	0.2	ESRD	*PAX2*	c.76dupG	p.Val26Glyfs*28	AD	P	[20]	up to 60

Pt, patient; WES, whole-exome sequencing; Ref, reference; AR, autosomal recessive; AD, autosomal dominant; ESRD, end-stage renal disease; CKD, chronic kidney disease; ACMG, American College of Medical Genetics and Genomics; LP, likely pathogenic; P, pathogenic; VUS, variant of unknown significance

^*^Nonsense mutation

^†^Variant was not found in NCBI (National Center for Biotechnology Information, https://www.ncbi.nlm.nih.gov), gnomAD (Genome Aggregation Database, https://gnomad.broadinstitute.org), UCSC Genome Browser (University of California, Santa Cruz Genome Browser, https://genome.ucsc.edu), or HGMD (The *Human Gene Mutation Database, *http://www.hgmd.cf.ac.uk)

### Family 1: Patient 1, 59-year-old woman

At the age of 51, patient (Pt) 1 started experiencing recurrent hyperuricemic attacks. After 2 years, the patient was diagnosed with renal insufficiency with persistent hyperuricemia, and febuxostat was prescribed for hyperuricemia. Eventually, renal insufficiency progressed to end-stage renal disease (ESRD) when the patient was 54 years old. The patient’s mother, brother, and elder sister all had hyperuricemia and ESRD, and required renal replacement therapy in their adulthood for ESRD. All the family members had no other underlying comorbidities, or hearing- or vision-related abnormalities. WES revealed the presence of a novel *UMOD* missense variant (NM_001008389.3: c.626G > T; p.Gly209Val). Thus, a diagnosis of autosomal dominant (AD) tubulointerstitial kidney disease-uromodulin-associated kidney disease (ADTKD-*UMOD*) (MIM 16,200; unknown prevalence) was made, and now, kidney transplantation from an unaffected offspring is planned for managing the patient’s condition.

### Family 2: Pt 2, 41-year-old man

Since the age of 29, Pt 2 suffered from recurrent flank pain. The patient was diagnosed with renal insufficiency associated with nephrolithiasis and left renal atrophy. The patient had undergone extracorporeal shock wave lithotripsy 13 times for the removal of a ureteral stone at the right ureteropelvic junction at the age of 30, and had undergone left nephrectomy at the age of 31. Importantly, the patient had no family history of renal insufficiency or nephrolithiasis. The pathology of the excised kidney was consistent with a diagnosis of xantho-granulomatous pyelonephritis. At the age of 39, the condition progressed to ESRD and the patient was transplanted with a kidney from his wife. Two months after transplantation, a biopsy of the allograft was performed as the serum creatinine level had not normalized (1.4–2.1 mg/dl) after transplantation. Pathological tests revealed the presence of intratubular calcium oxalate crystals along with tubular degeneration and atrophy. Primary hyperoxaluria type 1 was ruled out as no *alanine-glyoxylate aminotransferase* (*AGXT*) mutations were identified and oxalate excretion in urine was within the normal range. WES revealed the presence of a homozygous adenine phosphoribosyltransferase (*APRT)* nonsense variant (*NM_00485.2*: c.294G > A; p.Trp98*) [6]. After the diagnosis of APRT deficiency (MIM 614723, unknown prevalence), we initiated treatment with a xanthine oxidase inhibitor to prevent new stone formation and 2,8-dihydroxyadenine crystalluria to slowdown progressive renal insufficiency in the allograft.

### Family 3: Pt 3, 20-year-old woman

Pt 3 was the first child in the family. The patient had submucosal cleft palate with bifid uvula, low set ears, and long philtrum. Further, the patient suffered from recurrent otitis media with effusion, and speech delay with sensorineural hearing loss of both ears. The patient’s height remained within the 10th–25th percentile. At the age of 5, mild renal insufficiency (blood urea nitrogen, 11 mg/dl; creatinine, 0.6 mg/dl) was detected along with microscopic hematuria and proteinuria. Renal biopsy revealed diffuse global and segmental mesangiolysis. When the patient was 18-years old, bilateral small-sized kidney with nephrolithiasis had been diagnosed using renal ultrasonography (USG). Further, a diagnosis of chronic kidney disease (CKD) stage 2 was made based on an eGFR (estimated glomerular filtration rate) of 70.4 ml/min/1.73 m^2^ using the CKD EPI cystatin C calculation [7]. WES revealed the presence of a heterozygous *MAF* missense variant (NM_001031804.2: c.185C > T; pThr62Met), and a diagnosis of Ayme-Gripp syndrome (MIM 6010880, up to 21 patients reported worldwide) was made based on WES results and clinical signs, such as sensorineural hearing loss, distinctive flat facial appearance, skeletal anomalies, reduced growth, and renal involvement. The patient’s parents and healthy brother did not carry the variant. Evaluation for other systemic involvements is scheduled, and genetic counseling was provided for reproductive planning in the future.

### Family 4: Pt 4, 18-year-old boy

Pt 4 was the third child of nonconsanguineous Korean parents. The patient had had two elder sisters. At the age of 4, ESRD was identified when investigating the reason underlying poor oral intake and malaise. The patient underwent renal replacement therapy until undergoing cadaveric donor transplantation at the age of 18. In addition, skeletal dysplastic features including severe short stature and narrow thoracic cage were observed, as well as dense calvarium and tracheal and lower rib cartilage calcification. Cholestatic liver dysfunction— without evident etiology—with mild portal inflammation and bile ductular progression was diagnosed using liver biopsy at the age of 16. Ophthalmological examination revealed no abnormalities. WES revealed the presence of compound heterozygous *IFT140* variants NM_014714.3 (c.2650C > T; p.Arg884Trp) and NM_014714.3 (c.4309G > A; p.Glu1437Lys), resulting in a diagnosis of short-rib thoracic dysplasia 9 (SRTD 9) with or without polydactyly (MIM 266920, up to 20 patients reported worldwide). The patient’s eldest sister had been diagnosed with glomerulonephritis of unknown etiology at the age of 3, and had undergone cadaveric donor renal transplantation at the age of 7; she also exhibited similar skeletal features as the patient, carried identical variants, and had recurrent episodes of retinal detachment. Pt 5 is on regular follow up for ophthalmologic evaluation and monitoring for allograft and liver function. Notably, no variant was identified the patient’s mother, but his father and another unaffected sister were not tested.

### Family 5: Pt 5, 11-year-old boy

Pt 5 had renal insufficiency (serum creatinine 1.2–1.4 mg/dl) with proteinuria and hypertension at the age of 5. The growth profiles of the patient were normal, and he did not manifest any hearing or visual problems. When the patient was 11-years old, renal USG revealed that both kidneys were small sized (right kidney 7.6 cm, left kidney 6.9 cm) with poor cortico-medullary differentiation, increased echogenicity, and multiple renal cysts (< 8 mm in diameter). WES revealed a novel *PAX2* frameshift variant (NM_003990.5: c.124_139del; p.Val42Argfs*36), confirming the diagnosis of papillorenal syndrome (MIM 120,330, up to 60 patients reported worldwide). The patient’s parents and healthy sister did not carry the variant. Pt 6 is on a regular follow-up with visual and hearing checkups and supportive care for renal insufficiency. Genetic counseling was provided to the patient’s parents for reproduction planning in the future.

### Family 6: Pt 6, 10-year-old boy

At the age of 9, Pt 6 was diagnosed with asymptomatic proteinuria with renal insufficiency (CKD stage 2). Renal USG revealed small-sized kidneys (right kidney 8.0 cm, left kidney 6.4 cm) with increased echogenicity. The patient’s mother also had proteinuria with normal renal function and defective vision with uncertain etiology, but her hearing was normal. The patient’s maternal grandfather was on hemodialysis, and suffered from a hearing defect of unknown origin. Single gene testing for *CLCN5* and *OCRL* revealed no pathogenic variants. WES revealed the presence of a novel *PAX2* splice-site variant (NM_003990.5: c.617-1G > T). The patient’s mother also carried the variant, but his father and healthy brother did not carry the variant. Based on these results, a diagnosis of papillorenal syndrome (MIM 120330, up to 60 patients reported worldwide) with AD inheritance was made, and visual and hearing evaluation has been performed regularly with supportive care for renal insufficiency. Genetic counseling was provided for the family for future reproductive planning.

### Family 7: Pt 7, 9-year-old girl

Pt 7 had a urinary tract infection when she was 3-months old, and renal USG incidentally revealed medullary nephrocalcinosis of both kidneys. Hypercalciuria (urine calcium/creatinine ratio 0.57–0.72 mg/mg) with normal serum calcium level was detected, and renal function was normal. The patient’s growth profiles and development were normal. Panel gene tests for mutations associated with genetic kidney diseases (*GXT, CLCN5, CLDN16, CLDN19, CNNM2, CTNS, EGF, FXYD2, GRHPR, HNF1b, HOGA1, KCNA1, OCRL, SLC22A12, SLC2A9, SLC3A1, SLC5A2, SCL7A9, TRPM6,* and *VDR)* revealed no pathogenic variants. At age of 9, patient height and weight were in the 10th–25th percentile. Persistent nephrocalcinosis with hypercalciuria was investigated. WES revealed a homozygous *CYP24A1* missense variant (NM_000782.4: c.376C > T; p.Pro126Ser). Both parents were heterozygous carriers. After the diagnosis of hypercalcemia, infantile 1 (MIM 143,880, unknown prevalence), diet-related education was provided to avoid hypercalcemia.

### Family 8: Pt 8, 7-year-old girl

Pt 8 is the first child of Korean nonconsanguineous parents. The patient had two healthy younger twin brothers. At the age of 1 month, the patient developed transient cyanosis with seizure-like motion caused by hypocalcemia and hypomagnesemia. The patient had been prescribed a calcium and magnesium supplement without genetic assessment. She was referred to our medical genetic center at the age of 7 for further evaluation. The patient exhibited normal serum calcium levels with mild hypercalciuria (urine calcium/creatinine ratio 0.22–0.35 mg/mg), hypomagnesemia (1.3–1.6 mg/dl) with low urinary loss (urine magnesium/creatinine < 0.029 mg/mg), and normal renal function. Kidney USG showed no abnormal findings. The patient showed normal growth and developmental milestones. WES revealed compound heterozygous mutations in *TRPM6* [(NM_001177311.1: c.1421A > G; p. Tyr474Cys in exon 12 inherited from the mother and c.4917_4918delAA; p.Lys1639Asnfs*4 in exon 29 inherited from the father]. The patient’s two twin brothers are asymptomatic carriers of the missense variant c.1421A > G (p. Tyr474Cys). After the diagnosis of hypomagnesemia 1 intestinal (MIM 607009, unknown prevalence), magnesium replacement was increased with reduced calcium supplement based on the pathophysiology of secondary hypocalcemia due to primary hypomagnesemia.

### Family 9: Pt 9, 1-month-old girl

Right renal agenesis with left renal hypoplasia with ectopy was suspected in prenatal USG at a gestational age (GA) of 20 weeks. Due to the premature rupture of the membrane, an emergent caesarian section was performed for birth at GA 36 weeks. The delivery was uneventful. Postnatal renal USG on day 3 revealed small echogenic kidneys with multiple cysts, and azotemia progressed (maximal serum BUN 83.2 mg/dl, and creatinine 2.59 mg/dl), and peritoneal dialysis was started since day 17 after birth. WES revealed a frameshift *PAX2* variant (NM_003988.4: c.[69delinCG]; p.[Val26Glyfs*28]). The patient’s parents did not carry the variants. Regular surveillance has been done for possible ophthalmologic involvement.

### Pathogenicity of identified variants

**Fig. 1:**
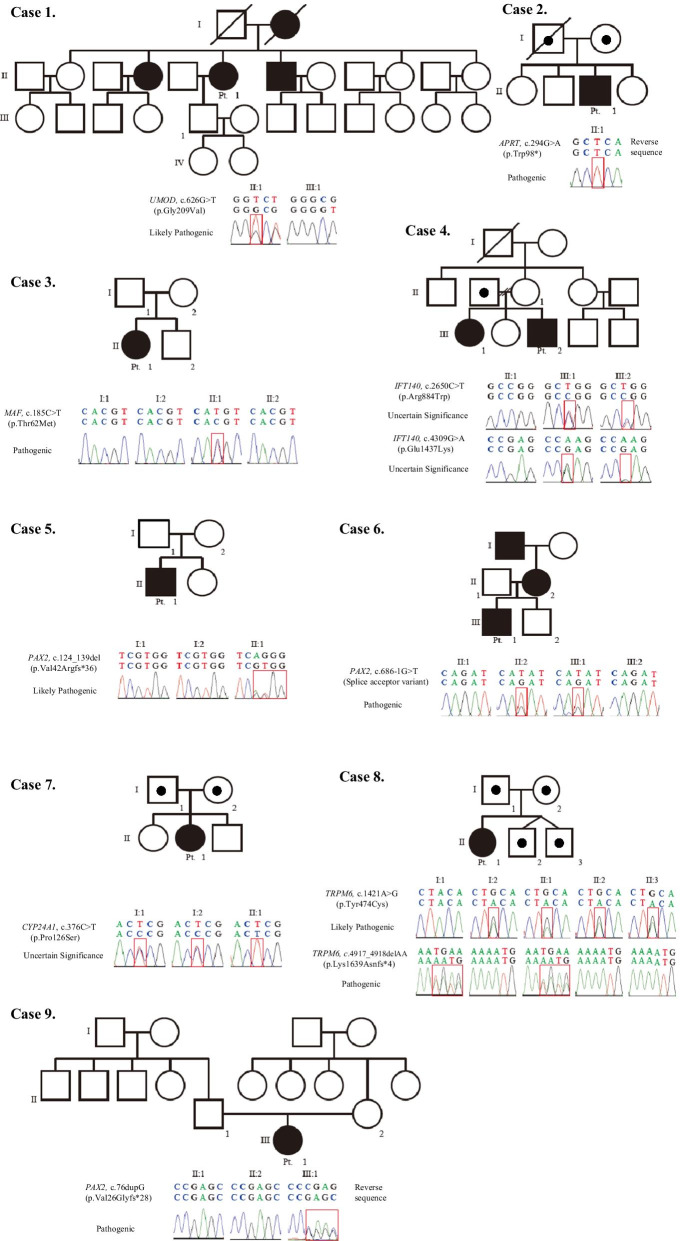
Pedigree of each patient and family with identified variants including family testing. Pedigree of 9 families are presented with each genetic alteration found from whole-exome sequencing and pathogenecity evaluated by American College of Medical Genetics and Genomics (ACMG) classification

Identified variants were analyzed and classified according to the American College of Medical Genetics and Genomics (ACMG) classification [8] and presented in Table 1. The pedigree of each patient is presented in Fig. 1 with the identified variants.

A total of 7 variants were previously unreported. Sanger sequencing was done for the identified variant in the patients and their family members. The *APRT* mutation in Pt 2, *TRPM6* mutation in Pt 8, and the *PAX2* mutation in Pt 5, 6 and 9 were classified as “pathogenic” before and after the family tests. The *MAF* mutation in Pt 3 was initially assessed as “likely pathogenic (LP),” but reassessed as “pathogenic (P)” after the family test. The *UMOD* mutation in Pt 1 was assessed as “LP,” before and after testing of her son. The *IFT140* mutations in Pt 4, and *CYP24A1* mutation in Pt 7 were classified as “variant of unknown significance (VUS).” Pt 2 had an additional missense variant of the *MYL3* gene, as a secondary incidental finding reported based on the ACMG guideline [8], NM_000258: c. 170C > G (p.Arg57Gly), with no clinical significance at the time of evaluation. There was no incidental finding, unrelated to patient’s main phenotypes, in the other 8 families.

## Discussion

The current report described the detailed clinical and genetic features of 7 ultra-rare diseases from 9 unrelated Korean families. These ultra-rare diseases are described in Table 1. Among these, 3 diseases have AD inheritance: ADTKD-*UMOD*, Ayme-Gripp syndrome due to *MAF* mutation, and papillorenal syndrome due to *PAX2* mutation. The other 4 diseases have autosomal recessive inheritance: APRT deficiency, SRTD 9 with or without polydactyly due to *IFT140* mutations, hypercalcemia infantile type1 with altered calcium metabolism due to *CYP24A1* mutations, and hypomagnesemia 1 intestinal or hypomagnesemia with secondary hypocalcemia due to *TRPM6* mutations.

WES was requested in each patient because of a positive family history (Pt 1, 4, and 6), the involvement of extra-renal organs (Pt 3 and 4), early-onset renal insufficiency (Pt 5, 6, and 9), persistent electrolyte imbalance (Pt 7 and 8), or the recurrence of original renal disease after transplantation (Pt 2). In particular, Pt 1 had a positive family history of renal insufficiency with suspicion of ADTKD spectrum disease [9]. Pt 5, 6, and 9 had developmental dysplasia of the kidney with renal insufficiency and or proteinuria. Pt 3 and 4 showed extra-renal manifestations such as skeletal dysplasia or facial dysmorphism with glomerulopathy.

Currently, targeted gene panel tests are widely used in clinics that are designed to screen the known 20–100 genes responsible for genetic renal diseases. The read depth of the target regions is higher in the panel gene test than in WES. However, ultra-rare diseases may not be included in the panel gene list, as in Pt 5–7, and 9; therefore, the time and expense for the genetic diagnosis might have been extended without WES.

Importantly, the diseases diagnosed by WES were sometimes unexpected. However, according to the “genotype to phenotype paradigm,” based on clinical information and the results from genome sequencing in parallel, each disease was validated as responsible for respective patient’s phenotypes. For example, Pt 3’s dysmorphic face, organ anomalies, and short stature led the physician to suspect RASopathy [10], and glomerulopathy was suspected as a separate disease. However, as MAF, a leucine zipper-containing transcription factor of the AP1 superfamily, is involved in the embryonic development of human lens, cochlear cells, dorsal spinal cord, dorsal root ganglia, skin, kidney, and hypertrophic chondrocytes of vertebrae, rib, and limb cartilages [11], its mutation is responsible for the full spectrum of the Pt 3’s phenotypes including skeletal, facial deformities, hearing defects, and renal involvement. A mutation from the same residue (c.185C > G; p.Thr62Met) has also been reported in the patient with similar phenotypes including cataract, deafness, intellectual disability, seizures, and Down syndrome-like faces, further supporting our finding [12]. SRTD 9 in Family 4 was another unexpected diagnosis; familial early-onset ESRD first raised the possibility of hereditary renal disease, but the short stature was attributed to the ESRD, and their inappropriate small thoracic cage was not clinically investigated. However, as Schmidts et al. suggested the expression of *IFT140* not only in renal and retinal tissues but also in the skeleton. *IFT140* deficiency is a rare cause of severe types of skeletal dysplasia from Jeune asphyxiating thoracic dystrophy with renal involvement with or without retinal involvement to nonlethal thorax-related clinical course with no polydactyly as in Pt 4 [13].

WES is most beneficial with respect to helping physicians in the following aspects: deciphering the pathophysiology of each disease; predicting the prognosis of the affected patient; providing an appropriate alternative intervention; delaying disease progression; and, if possible, treatment (Table 2.). Notably, in Pt 2 with APRT deficiency, excessive production and renal excretion of 2,8-dihydroxyadenine was the key pathophysiology leading to the recurrent formation of renal stones and eventual renal insufficiency with tubular degeneration. Only early recognition, use of xanthine oxidase inhibitors, and dietary purine restriction can help preserve renal function, even if renal insufficiency has progressed [14]. Moreover, recurrent 2,8- dihydroxyadenine-induced nephropathy can develop in the engrafted kidney in affected patients, sometimes progressing to recurrent renal insufficiency. Therefore, the use of xanthine oxidase inhibitors should be maintained throughout the patient’s life even after kidney transplantation [14–16]. Accordingly, Pt 2 was initiated on allopurinol at the age of 41, immediately after definitive diagnosis and 18 months after kidney transplantation, to preserve renal allograft function (hopefully). For Pt 8, pharmacological treatment was changed after the detection of *TRPM6* variants—i.e., calcium replacement was discontinued and magnesium supplement was increased—because the severe magnesium deficiency caused hypocalcemia secondary to the impaired synthesis and secretion of the parathyroid hormone [17]. Treatment options for renal transplantation were also affected based on the results of genetic diagnosis and family testing, as in case of Pt 1; the donor for kidney transplantation was selected after the affected renal disease was ruled out in the potential donor for Pt 1.

**Table 2:** Clinical impact of genetic diagnosis using WES

Pt No	Purpose of WES	Diagnosis	Clinical impact
1	Evaluation for familial disease for making a decision regarding kidney donation from a family member	Autosomal dominant tubulointerstitial disease-Uromodulin-associated kidney disease	Evaluation of other family members, plan for kidney donation from unaffected son, justification of the use of uric acid lowering agent
2	Anticipation for recurrent disease after kidney transplantation	APRT deficiency	Genetic counseling, start medication retarding renal damage due to underlying disease
3	Evaluation for possible underlying etiology for syndromic disease	Ayme-Gripp syndrome	Genetic counseling for reproductive plan, surveillance for other organ involvement including cataract, brain imaging, skeletal abnormalities, endocrine function, gastrointestinal function
4	Evaluation for possible underlying etiology for familial syndromic disease	Short-rib thoracic dysplasia 9 with or without polydactyly	Genetic counseling, surveillance for other organ involvement including hepatic/retinal involvement
5	Evaluation for underlying etiology for incidental childhood CKD progression	Papillo-renal syndrome	Genetic counseling for reproductive plan, evaluation of other family members, surveillance for other organ involvement including vision and hearing
6	Evaluation for underlying etiology for incidental childhood CKD progression	Papillo-renal syndrome	Genetic counseling for reproductive plan, evaluation of other family members, surveillance for organ involvement including vision and hearing
7	Evaluation for underlying etiology for neonatal nephrocalcinosis	Hypercalcemia, infantile, 1	Genetic counseling for reproductive plan, establishment of preventive principles for aggravation of the signs and symptoms
8	Evaluation for underlying etiology for neonatal persistent severe hypomagnesemia, hypocalcemia	Hypomagnesemia 1, intestinal	Genetic counseling for reproductive plan, establishment of long-term management plan based on pathophysiology
9	Evaluation for congenital renal disease leading to ESRD	Papillo-renal syndrome	Genetic counseling for reproductive plan, evaluation of other family members, surveillance for other organ involvement including vision and hearing

Pt, patient; WES, whole-exome sequencing; APRT, adenine phosphoribosyltransferase; CKD, chronic kidney disease; ESRD, end-stage renal disease

Genetic diagnosis helped us understand the systemic constellation of symptoms and mutations associated with each disease and to lookout for extra-renal organ phenotypes in the affected patients including opthalmological evaluations for Pt 5 and Pt 6 harboring a *PAX2* mutation [18–20]; neurological evaluations including brain imaging, ophthalmic evaluation including cataract, endocrinal assessment and skeletal survey for Pt 3 with a *MAF* mutation [10]; and examination for hepatic dysfunction, intraocular abnormality, and progression of skeletal dysplasia for Pt 4 and his affected sister harboring *IFT140* mutations [21].

It is accepted that a large portion of ultra-rare diseases may be caused by de novo mutations. They are generally more deleterious than inherited variation because they have been subjected to less stringent evolutionary selection [22]. On average, as 74 germline single-nucleotide variants (SNVs), 3 indels, and 0.02 de novo copy number variants (CNVs) occur in one person’s genome, one de novo mutation can develop per exome [23]. For disorders caused by particular variants in a single gene or monogenic disorders, as in our study, the low probability of mutational event renders these disorders extremely rare in the population. However, as there are factors which do increase the intrinsic propensity for de novo mutations, such as high CpG density leading to increased rates of de novo SNVs, and segmental duplications leading to increased rates of de novo CNVs [24, 25], these ultra-rare diseases would be observed more frequently than expected.

Family member screening is important to verify de novo mutations in diseases with AD inheritance or compound heterozygosity in diseases with autosomal recessive inheritance. In addition, as in Pt 3, family testing assesses the pathogenicity of a variant. Identification of unrevealed but affected family members is another aspect that would enable the use of appropriate management strategies to improve the clinical outcomes of unrecognized renal diseases, as in family 6. The diagnosis of Pt 4 is controversial; identical variants were identified in the elder sister, who also manifested a similar phenotype, but no variant was identified in the patient’s mother. Although family testing was incomplete, we assumed that the patient’s father could be the carrier of one mutated allele, and the mother might have passed the other mutated allele through germline mosaicism.

As patients or other family members enter the reproductive age, counseling should be provided regarding the inheritance of the disease, and the need for prenatal or preimplantation genetic diagnosis during future reproductive planning, a strategy that was employed in case of most of the families in this study (Table 2). Perceiving the need for sibling testing and providing appropriate counseling regarding the possibility of any offspring inheriting the condition should also be highlighted.

## Conclusion

WES is an effective tool to identify ultra-rare genetic renal diseases. By facilitating diagnosis, WES helps us better understand ultra-rare diseases and provides a roadmap to establishing appropriate counseling, surveillance, and management strategies. Increasing information regarding ultra-rare genetic diseases—with more cases being reported across the globe—would result in the development of more disease-specific management strategies aimed at ensuring optimal patient care.

## Data Availability

All data supporting our results are included in this published article. The raw data of whole-exome sequencing of the patient in this study are not publicly available in order to protect participant confidentiality, but are available from the corresponding author on reasonable request. If you want to request access to the data, please contact professor BH Lee at the Department of Pediatrics in Asan Medical Center Children's hospital, Seoul, Korea.

## Abbreviations

WES: Whole-exome sequencing
USG: Ultrasonography
Pt: Patient
CKD: Chronic kidney disease
ESRD: End-stage renal disease
ADTKD: Autosomal dominant tubulointerstitial kidney disease
eGFR: Estimated glomerular filtration rate
ACMG: American College of Medical Genetics and Genomics
P: Pathogenic
LP: Likely pathogenic
VUS: Variant of unknown significance
